# Combination of Dietary Fibers From Different Food Origins as a Treatment for Adults With Functional Constipation: A Randomized Clinical Trial

**DOI:** 10.1002/fsn3.72048

**Published:** 2026-06-26

**Authors:** Alessandro Di Minno, Maria Vittoria Morone, Daniele Giuseppe Buccato, Lorenza Francesca De Lellis, Hammad Ullah, Angela Cerqua, Roberto Piccinocchi, Costanza Riccioni, Danaè Larsen, Alessandra Baldi, Antonietta di Guglielmo, Gaetano Piccinocchi, Hesham R. El‐Seedi, Roberto Sacchi, Maria Daglia

**Affiliations:** ^1^ Department of Pharmacy University of Napoli Federico II Naples Italy; ^2^ CEINGE‐Biotecnologie Avanzate Naples Italy; ^3^ School of Pharmacy, University of Management and Technology Lahore Pakistan; ^4^ Level 1 Medical Director Anaesthesia and Resuscitation A. U. O. Luigi Vanvitelli Naples Italy; ^5^ R&D Department Esserre Pharma Srl Rome Italy; ^6^ School of Chemical Sciences The University of Auckland Auckland New Zealand; ^7^ Comegen S.c.S., Società Cooperativa Sociale di Medici di Medicina Generale Naples Italy; ^8^ International Research Center for Food Nutrition and Safety Jiangsu University Zhenjiang China; ^9^ Chemistry Department, Faculty of Science Islamic University of Madinah Madinah Saudi Arabia; ^10^ Applied Statistic Unit, Department of Earth and Environmental Sciences University of Pavia Pavia Italy

**Keywords:** dietary fiber, functional constipation, oat fiber, pectin, randomized clinical trial, resistant dextrin

## Abstract

Laxatives are the primary treatment for functional constipation (FC), but they are not always effective and well‐tolerated. Dietary fiber can represent a valid alternative. This study aimed to determine whether combining dietary fibers from different origins could be a valuable strategy for FC. Thus, the efficacy of a fiber‐based formulation (MNS‐36), composed of resistant dextrin from wheat starch ( *Triticum aestivum* L.), pectin, and insoluble fibers from *Citrus* spp., and oat fibers ( *Avena sativa* L.) was tested in a monocentric, double‐blind, randomized, placebo‐controlled clinical trial with 54 adults with FC. Participants received two sachets of MNS‐36 or a placebo daily for 28 days, with weekly visits and a follow‐up. Compared to the placebo group, bowel movements, used as the primary outcome of the study, increased significantly between baseline and week 4 (*β* = 2.43 ± 0.23, *p* < 0.001). Similarly, Bristol Stool Form Scale scores significantly improved in the treatment group (*p* < 0.001), being normalized from week 2 onwards (*p* ≤ 0.008). In the treatment group, significant reductions were also observed in abdominal bloating (*β* = 1.21 ± 0.17, *p* < 0.001), abdominal distension (*β* = 1.66 ± 0.18, *p* < 0.001), heaviness (*β* = 1.39 ± 0.21, *p* < 0.001), and flatulence (*β* = 1.51 ± 0.26, *p* < 0.001), whereas abdominal pain did not differ significantly between groups. No adverse events were reported, and treatment adherence was high. These findings highlight the efficacy of combining soluble and insoluble fibers from different food origins in improving gut function, addressing both symptoms and tolerability, and underscore this strategy as a natural alternative approach to laxatives for managing FC.

## Graphical abstract

This randomized, double‐blind, placebo‐controlled trial evaluated the efficacy of MNS‐36, a combination of soluble and insoluble dietary fibers, in adults with functional constipation. After 28 days, MNS‐36 significantly improved bowel movement frequency, stool consistency, and gastrointestinal symptoms, including bloating and abdominal distension, compared with placebo. The treatment was well tolerated, with no adverse events reported, supporting its potential as a safe, natural alternative to laxatives.

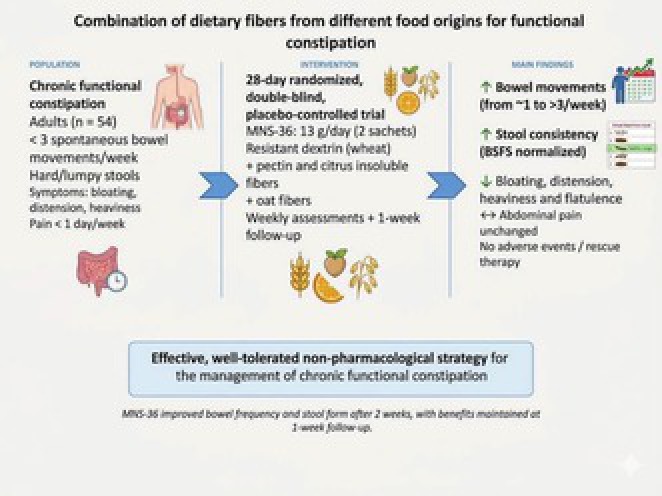

## 1 Introduction

Constipation is defined as having fewer than three bowel movements per week. It also encompasses irregular bowel movements, hard or lumpy stools, excessive straining, and a sensation of incomplete evacuation or blockage (Vriesman et al. 2020). Symptoms can be acute, lasting less than a week, typically triggered by dietary or lifestyle changes, or chronic, persisting for at least 3 months. Chronic constipation affects 10%–15% of the general population, making it one of the most common gastrointestinal disorders. It significantly impacts quality of life, imposes a considerable burden on healthcare, and often requires both primary and secondary care (Arco et al. 2022). According to the Rome IV criteria, individuals who have experienced chronic constipation for at least 3 months without any underlying organic gastrointestinal disease are categorized into four subtypes: (a) functional constipation, (b) irritable bowel syndrome with constipation, (c) opioid‐induced constipation, and (d) functional defecation disorders, including inadequate defecatory propulsion and dyssynergic defecation (Schmulson and Drossman 2017). The initial treatment approach for these conditions is essentially uniform. It emphasizes adequate hydration (at least 2 L of water daily) and dietary modifications (such as the consumption of whole grains, legumes, vegetables, and fruits). As long‐term adherence to a high‐vegetable diet is generally low and the increase in the vegetable intake may also cause side effects such as gas, bloating, and abdominal distension (Pannemans et al. 2020), the subjects resort to the use of over‐the‐counter laxatives (Shin et al. 2019), which are not free from adverse effects. Thus, many individuals with functional constipation report incomplete relief or difficulty maintaining adherence to these treatments. This underscores the need for effective and well‐tolerated alternative treatments.

Dietary fiber offers a potential solution by combining natural bioactive compounds, addressing both symptom relief and tolerability (Liu, Asif, et al. 2024). Dietary fiber, which is not digested in the small intestine, can either be fermented by gut bacteria or contribute to bulk formation in the colon, promoting water retention (Rao et al. 2015). Systematic reviews suggest that soluble fiber is more effective than insoluble fiber in alleviating constipation symptoms (Suares and Ford 2011). A low fiber intake, typically around 7 g/day, can reduce stool frequency, thus a gradual increase to 30 g/day is recommended.

*Triticum aestivum* L. (family Poaceae), commonly referred to as wheat, is among the most widely consumed cereal crops worldwide, alongside rice and maize, due to its versatility and broad range of applications. Over recent years, global wheat production has been largely dominated by countries such as China, India, Russia, the United States, and Canada (Limbore 2015; Zhao, Zhai, et al. 2019). Beyond its central role in human nutrition, wheat also contains bioactive components with potential physiological effects (Zhao, Shi, et al. 2019). Among these, resistant dextrin—also known as indigestible dextrin—is a soluble dietary fiber obtained from wheat starch through controlled processing steps, including dextrinization and subsequent depolymerization. These modifications generate atypical glycosidic linkages, such as α‐1,2 and β‐1,6, which contribute to its resistance to enzymatic digestion (Han et al. 2018; Hobden et al. 2015; Li et al. 2023; Trithavisup et al. 2019). Structurally, resistant dextrin is characterized by the presence of hydrophilic groups and a specific molecular arrangement that underlies its functional properties, including water‐binding capacity, viscosity, and stability. Due to its limited digestibility, it persists longer within the gastrointestinal tract. Similar to other dietary fibers, it contributes to bowel regularity and may enhance satiety. In addition, resistant dextrin exerts prebiotic effects by promoting the growth of beneficial gut microbiota and supporting intestinal homeostasis. It has also been associated with metabolic benefits, including reduced glucose absorption and modulation of lipid metabolism, partly through decreased cholesterol absorption and bile acid reabsorption in the small intestine (Aliasgharzadeh et al. 2015; Ye et al. 2015).

Citrus (family Rutaceae) is one of the most widely cultivated fruit types worldwide. The processing of citrus fruits generates significant agrifood residues, with peels accounting for 50%–60% of the total output. This results in over 540,000 tons of agrifood residues annually, contributing to environmental pollution and resource depletion. Interestingly, citrus peel, which is traditionally used in cooking to prepare candied sweets, contains up to 50% more dietary fiber than the fruit itself, drawing the attention of researchers and the food industry to its potential for extraction and the applications. Recent studies emphasize the relationship between the structural properties of citrus fiber and its biological activities (Liu, Wang, et al. 2024). Among these are pectins, which have broad applications in the pharmaceutical and food industries due to their beneficial properties. Pectin mainly consists of galacturonic acid, rhamnose, galactose, arabinose, fructose, and xylose (Munarin et al. 2012). It is recognized as a safe dietary ingredient for treating constipation and is particularly effective for individuals with reduced intestinal motility, dry stool, and painful defecation. In the colon, pectin absorbs water, a process driven by hydrogen bonding, causing it to swell and increase the volume of intestinal contents. This swelling provides a lubricating effect that accelerates the defecation process (Rtibi 2018). Experimental studies have shown that gastric‐perfused pectin enhances the water‐retaining capacity of feces in mice, increasing water content by up to 26% and demonstrating significant anti‐constipation effects in vivo (Li, Zhu, et al. 2019; Li, Jin, et al. 2019).

*Avena sativa* L. (family Poaceae), commonly known as oat, stands out among cereal crops due to its diverse nutritional profile and versatility in human food, animal feed, healthcare, and cosmetics. An annual crop cultivated for over 2000 years, oats are one of the oldest crops known to humanity, originating several thousand years after cereals like wheat and barley. Oats are also rich in soluble fibers (Paudel et al. 2021). According to the United States Department of Agriculture, 100 g of whole‐grain oats contains 10.4 g of dietary fiber, 7.52 g of β‐glucan, and 53.8 g of starch (Laboratory 2025). These components contribute significantly to the health benefits associated with oat consumption, making it a valuable cereal crop for the food industry.

Despite the well‐documented benefits of soluble and insoluble fibers in maintaining proper intestinal function, to our knowledge, no clinical trials have evaluated the synergistic effects of the combination of dietary fibers from different origins, each with distinct mechanisms of action, in subjects with functional constipation for the improvement of bowel function and overall gastrointestinal well‐being. Thus, to address this gap, we conducted a monocentric, randomized, double‐blind, placebo‐controlled clinical trial to evaluate the efficacy and tolerability of a fiber‐based formulation (MNS‐36) composed of resistant dextrin from wheat starch ( *Triticum aestivum* L.), pectins and insoluble fibers from *Citrus* spp., and oat fibers ( *Avena sativa* L.) in individuals with chronic functional constipation.

## 2 Materials and Methods

### 2.1 Dietary Fiber‐Based Formulation and Placebo

This clinical trial investigated a dietary fiber‐based formulation (hereafter referred to as MNS‐36) consisting of resistant dextrin from wheat starch, pectins and insoluble fibers from *Citrus* spp., and oat fibers ( *Avena sativa* L.). The fiber blend consisted of resistant dextrins, pectins, and oat‐derived fibers. Each 6.5 g serving provided 5 g of dietary fiber, approximately 80% of which was soluble fiber and 20% insoluble fiber. The soluble fraction comprised resistant dextrins, pectins, and β‐glucans naturally present in oat fiber, whereas the insoluble fraction consisted primarily of cellulose and non‐cellulosic polysaccharides, mainly arabinoxylans and hemicelluloses, derived from oat and citrus sources. Both MNS‐36 and the placebo were produced by Esserre Pharma Srl. (Rome, Italy), in accordance with European regulations on contaminants and microbiological safety and were provided free of charge for the study. MNS‐36, notified to the Italian Ministry of Health (notification number: 168617), contained resistant dextrin from partially hydrolyzed wheat starch ( *T. aestivum* ), oat fiber ( *A. sativa* ), pectin, isomalt, orange flavor, orange fiber (*Citrus* spp.), silicon dioxide, and sucralose. The placebo, indistinguishable in appearance and flavor, included maltodextrin, isomalt, orange flavor, silicon dioxide, sucralose, and caramel color.

Sachet net weight (6.5 g) was controlled using Metrostat statistical software, adhering to Italian Law 25 October 1978, n. 690, and UNI ISO 2859 guidelines. Upon arrival at the clinical trial center, shipments were registered and packing slip details (batch numbers, manufacturing and expiry dates, manufacturer, quantity, and storage conditions) were verified. The MNS‐36 and placebo were stored at controlled room temperature (20°C–25°C) in a locked cabinet within a locked room, with maintained and regularly reviewed entry/exit and accountability logbooks. Each sachet was dissolved in 200 mL of water, thoroughly mixed, and administered orally twice daily (daily dose of 13 g).

### 2.2 Clinical Trial Design

A single‐center, randomized, double‐blind, placebo‐controlled, parallel‐group clinical trial was performed by COMEGEN Soc. Coop. Sociale (Naples, Italy) to evaluate the effects of MNS‐36, a dietary fiber‐based formulation composed of resistant dextrins derived from wheat starch, fibers from *Citrus* spp. and oat, on symptom improvement in individuals with chronic functional constipation. The study was double‐blind, both for the investigators and participants. For this purpose, both the dietary fiber‐based formulation and the placebo were made unrecognizable in shape, weight, color, and taste.

The participants received oral and written information regarding the study before they gave their written consent. The trial was registered with ISRCTN with ID number 45695355 (https://www.isrctn.com/ISRCTN45695355, accessed 1 October 2025) and conducted in accordance with the 1964 Declaration of Helsinki (as revised in 2000), approved by the Campania 1 Ethics Committee (protocol no. 134, 13 May 2024—N. Reg. 1–2024). The protocol and letter of intent received Ethics Committee approval.

The study design follows the suggestions reported on the EFSA guidance on the scientific requirements for health claims related to the gastrointestinal tract for evaluating the clinical efficacy of commonly used food components or supplements on bowel function. (EFSA Panel on Dietetic Products and Allergies 2016) It included two experimental groups, with 54 participants randomly assigned in a 1:1 allocation ratio by means of simple randomization (27 participants, each group). Participants consumed two sachets daily (treatment or placebo) for 28 days, undergoing six visits: baseline (T0), and weekly for 4 weeks (T1‐T4), and a week‐long post‐treatment follow‐up visit (T5). As the study included two groups, group 1 (TREAT group) participants took the dietary fiber‐based treatment, and group 2 (CTR group) participants took a placebo. Both treatments were administered simultaneously, with parallel response variables measured at the start of treatment (T0), at 1‐weekly intervals (T1, T2, T3, T4), and after 1 week of follow‐up (T5). To assess the treatment adherence, residual sachets were counted at visits t1, t2, t3 and t4. The protocol of the study was registered on 19/11/2024 (https://www.isrctn.com/ISRCTN45695355).

To minimize bias and to ensure consistent results with the final aim of reproducing as best as possible the real conditions of use of the dietary fiber‐based formulation, participants were instructed not to alter their usual diet before or during the study. In addition, they were provided with guidelines on proper defecation posture (a pictogram was given at T0 (Irvine et al. 2016)), the recommended daily fluid intake of two liters, which was recorded in the Bowel Function Diary and the requirement to document any pharmacological treatments taken, which were recorded at each visit.

#### 2.2.1 Outcomes of the Study

The primary outcome of the present clinical study was to evaluate the change in the number of spontaneous complete bowel movements per week (bm/w). A bowel movement was classified as spontaneous and complete if it occurred naturally, without the use of any laxatives (including medications, over‐the‐counter remedies, dietary supplements, enemas, or suppositories) within the 24 h preceding the bowel movement. Data collection involved a baseline assessment of the average number of bm/w in the preceding month (obtained through a structured interview at the initial visit, T0), followed by daily completion of a bowel function diary (recording the number of bowel movements, time, and stool consistency using the Bristol Stool Form Scale (BSFS)) throughout the 28‐day intervention period. Weekly clinic visits (T1‐T4) provided an additional opportunity to confirm bm/w counts reported in the diary. A final report of bm/w for the preceding week was obtained during a post‐treatment follow‐up visit (T5).

Secondary outcomes evaluated the stool consistency (measured by the BSFS, using data from the daily diary and clinic visits), the intensity of commonly reported constipation symptoms (abdominal bloating/distension, heaviness, pain, flatulence, and difficulty passing stools, rated on a 0–5 visual analog scale (VAS), daily in the diary and verified during visits) and the use of any salvage treatments (laxatives or other remedies used to induce bowel movements).

#### 2.2.2 Study Population

Participants were recruited at the clinical trial site (COMEGEN Soc. Coop. Sociale, Naples, Italy) among outpatients referred by general practitioners or presenting spontaneously with symptoms of chronic functional constipation. Eligible individuals were screened according to predefined inclusion and exclusion criteria. As far as inclusion criteria are concerned, participants including both sexes, aged 18–70 years and capable of understanding and providing informed consent were included. All participants were HIV‐negative (rapid saliva test) and (where applicable) returned negative pregnancy tests. Inclusion criteria included chronic constipation symptoms (≥ 3 months, onset ≥ 6 months prior) with infrequent abdominal pain (< 1 day/week), < 3 bm/wk., and at least one of the following: straining during > 25% of defecations, lumpy/hard stools (Bristol types 1 or 2) in > 25% of movements, incomplete evacuation in > 25%, anorectal obstruction/blockage in > 25%, or manual evacuation maneuvers in > 25% of defecations. Exclusion criteria were: age < 18 or > 70 years, pregnancy/breastfeeding, abdominal pain ≥ 1 day/week (typical of irritable bowel syndrome), organic intestinal diseases, gastrointestinal surgery history, gastroesophageal reflux, Parkinson's/Alzheimer's disease, opioid or bowel‐function‐affecting medication use, antibiotic use (within 4 weeks), concurrent medication use (excluding over‐the‐counter analgesics), alcohol/drug/caffeine/theine addiction, cognitive impairment hindering questionnaire response, allergy to MNS‐36 ingredients, and/or acquired HIV immunodeficiency. The final sample comprised 54 participants (25 women, 29 men), 27 in each group (TREAT and CRT groups).

#### 2.2.3 Safety and Tolerability

All MNS‐36 ingredients comply with European food regulations and are considered safe. While no adverse events were anticipated, participants were monitored continuously. Suspected adverse reactions were reported via the VigiErbe online phytovigilance system (www.vigierbe.it; Accessed 11 December 2023), following Istituto Superiore di Sanità guidelines. Suspected Unexpected Serious Adverse Reactions (SUSARs) were reported in writing to the Ethics Committee. Subjects with sensitivity, intolerance, or allergy to dietary fibers were categorically excluded from the study.

### 2.3 Statistical Analysis

Sample size calculations were conducted using three power levels (1‐β) of 0.80, 0.95, and 0.99, with a significance level (α) set at 0.05, and three effect size values (Cohen's *f* = 0.10, 0.25, and 0.40, respectively). The within‐subject correlation value was fixed at *r* = 0.5, assuming there is a moderate correlation between measurements within the same participant. In a precautionary scenario, with an estimated drop‐out of 20% of the study population, it was decided to enroll a total of 54 subjects, (two groups of 27 subjects each).

The randomization sequence was created by a statistician using STATA 16 software (Stata Statistical Software: Release 16, College Station, Texas: StataCorp LLC). This sequence was stored in opaque, sealed envelopes that were numbered sequentially and kept in a locked cabinet at the experimental center. The physician responsible for enrolling participants was unaware of the assignment sequence throughout the study, ensuring that the treatment group's assignments were random and unpredictable. This method effectively reduced selection bias by minimizing systematic differences in baseline characteristics—such as prognostic factors or potential variations in treatment responses—between the groups.

To analyze how the experimental subjects responded to the treatments, we used linear mixed models with a random intercept (LMM). In these models, the time of measurement (T0 to T5), the treatment (control, CTRL, and treatment, TREAT), and their interaction were the fixed effects, while the identity of the subjects was the random effect to account for individual variability in response to the treatments. The sex and age (standardized to zero mean and unit standard deviation) of the experimental subjects were also added to the model as fixed effects. The seven response variables (i.e., bm/w, BSFS, and Symptoms A–E) were the dependent variable, each in a different model. Analyses were performed using the lme4 package (Bates et al. 2014) in R ver. 4.0.1 (Team 2022), and unless otherwise stated, data are reported as means ± standard deviation.

## 3 Results

**FIGURE 1:**
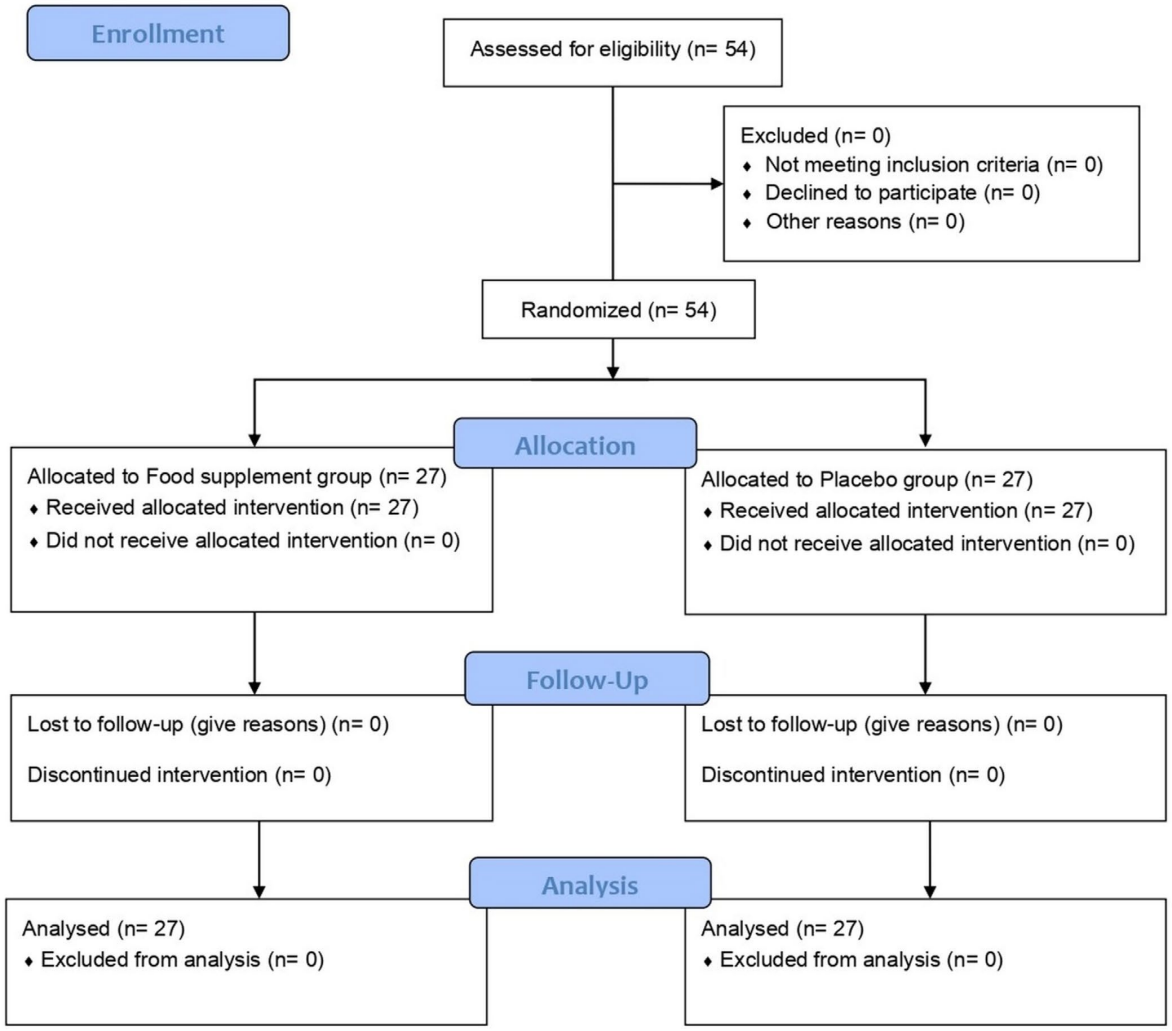
CONSORT flow diagram.

The study flow chart, produced in accordance with CONSORT PRO reporting guidelines (Calvert et al. 2013), is shown in Figure 1.

The two groups consisted of 54 subjects (27 subjects each) with a total of 29 males (corresponding to 53.7%, *n* = 14 of which were allocated to TREAT group) and 25 females (corresponding to 46.3%, *n* = 13 of which were allocated to group 1), both groups were administered treatments twice daily for 28 days. TREAT Group was treated with the formulation comprising fibers from wheat starch, *Citrus* spp., and oat at the daily dose of 13 g, while CRT group was treated with the placebo. The participants in each group had similar sociodemographic characteristics and clinical data, with no significant differences. The baseline characteristics of the subjects of each group are summarized in Table 1.

**TABLE 1:** Demographic data of the study population at baseline (T0).

Characteristics of the enrolled subjects	TREAT group (*n* = 27) dietary fiber‐based formulation	CRT group (*n* = 27) placebo
Median age (years)	49.7 ± 16.0	48.5 ± 17.0
Gender		
Male	*n* = 14 (49.5 ± 18.5 years)	*n* = 15 (52.7 ± 15.6 years)
Female	*n* = 13 (50.2 ± 10.9 years)	*n* = 12 (48.5 ± 11.8 years)
Ethnicity: Caucasian	27	27

Table 2 shows the descriptive statistics for the comparison between the placebo group (CRTL) and the dietary fiber‐based formulation treated group (TREAT) at T0‐5 for the number of bowel movements per week (bm/w), the score of Bristol Stool Form Scale (BSFS), and the gastrointestinal symptoms (Symptom A–E). Moreover, fluid intake data collected during the week preceding each study visit, which did not differ significantly between groups (*p* > 0.05), were reported.

**TABLE 2:** Descriptive statistics (mean, standard deviation, and range of values) for response variables measured at times T0–T5 in the two experimental groups.

Variables	TREAT–dietary fiber formulation						CRTLPlacebo					
	T0	T1	T2	T3	T4	T5	T0	T1	T2	T3	T4	T5
bm/w	1.2 ± 0.8 (0–2)	1.6 ± 0.5 (1–2)	2.7 ± 0.8 (1–4)	3.2 ± 0.8 (2–5)	3.6 ± 1.1 (1–5)	3.4 ± 0.9 (1–4)	1.1 ± 0.7 (0–2)	1.6 ± 0.6 (1–3)	1.6 ± 0.7 (0–3)	1.9 ± 0.9 (0–3)	1.8 ± 0.8 (0–3)	1.4 ± 0.6 (1–3)
BSFS	1.6 ± 0.5 (1–2)	1.7 ± 0.6 (1–3)	2.4 ± 0.6 (1–3)	3.3 ± 0.7 (2–4)	3.6 ± 1.0 (2–5)	3.2 ± 0.8 (2–4)	1.6 ± 0.5 (1–2)	1.8 ± 0.6 (1–3)	1.8 ± 0.5 (1–3)	1.9 ± 0.7 (1–3)	1.9 ± 0.7 (1–3)	1.8 ± 0.6 (1–3)
Symptom A	4.3 ± 0.5 (4–5)	4.4 ± 0.5 (4–5)	4.2 ± 0.7 (3–5)	3.6 ± 0.5 (3–4)	3.1 ± 0.4 (2–4)	3.2 ± 0.4 (3–4)	3.9 ± 0.8 (3–5)	4.2 ± 0.7 (3–5)	3.9 ± 0.7 (3–5)	4.0 ± 0.7 (3–5)	3.9 ± 0.8 (3–5)	4.0 ± 0.6 (3–5)
Symptom B	4.5 ± 0.5 (4–5)	4.3 ± 0.7 (3–5)	4.0 ± 0.6 (3–5)	3.5 ± 0.5 (3–4)	2.9 ± 0.5 (2–4)	3.2 ± 0.6 (2–4)	4.0 ± 0.7 (3–5)	4.1 ± 0.7 (3–5)	4.0 ± 0.8 (3–5)	3.8 ± 0.8 (3–5)	3.6 ± 0.7 (3–5)	3.6 ± 0.6 (3–5)
Symptom C	4.6 ± 0.5 (4–5)	4.2 ± 0.9 (1–5)	4.2 ± 0.4 (4–5)	3.6 ± 0.5 (3–4)	3.2 ± 0.7 (2–4)	3.2 ± 0.6 (2–4)	4.1 ± 0.9 (3–5)	4.3 ± 0.7 (3–5)	4.2 ± 0.7 (3–5)	3.8 ± 0.8 (3–5)	3.8 ± 0.8 (3–5)	3.8 ± 0.8 (3–5)
Symptom D	1.2 ± 0.4 (1–2)	2.0 ± 0.8 (1–3)	1.7 ± 0.6 (1–3)	1.5 ± 0.5 (1–2)	1.4 ± 0.5 (1–2)	1.7 ± 0.8 (1–3)	1.5 ± 0.5 (1–2)	1.7 ± 0.7 (1–3)	1.9 ± 0.9 (1–4)	1.5 ± 0.8 (1–4)	1.6 ± 0.7 (1–3)	1.9 ± 0.7 (1–3)
Symptom E	4.0 ± 0.8 (3–5)	3.5 ± 0.6 (2–4)	3.5 ± 0.5 (3–4)	3.0 ± 0.7 (2–4)	2.5 ± 0.6 (2–4)	2.5 ± 0.7 (2–4)	2.5 ± 1.1 (1–5)	2.7 ± 1.0 (1–4)	3.4 ± 1.3 (1–5)	3.5 ± 0.9 (2–5)	3.5 ± 0.9 (2–5)	3.2 ± 1.0 (1–5)
Fluid intake (L)	1.0 ± 0.2 (1.0–1.5)	1.1 ± 0.1 (1.0–1.4)	1.0 ± 0.2 (1.0–1.5)	1.0 ± 0.3 (1.1–1.5)	1.10 ± 0.2 (1.0–1.5)	1.2 ± 0.2 (1.0–1.5)	1.1 ± 0.4 (1.0–1.5)	1.0 ± 0.2 (1.0–1.3)	1.1 ± 0.2 (1.0–1.4)	1.0 ± 0.2 (1.0–1.5)	1.0 ± 0.2 (1.1–1.5)	1.0 ± 0.1 (1.0–1.5)

*Note:* bm/w (Primary end‐point): the frequency of evacuation following treatment with MNS‐36 or placebo; BSFS (Secondary outcome—Bristol Stool Form Scale score); Symptoms **A**: abdominal bloating, **B**: abdominal distension, **C** heaviness, **D** abdominal pain, and **E** flatulence, measured with an increasing rank scale from 0 to 5 (secondary endpoint).

### 3.1 Primary Outcome

**FIGURE 2:**
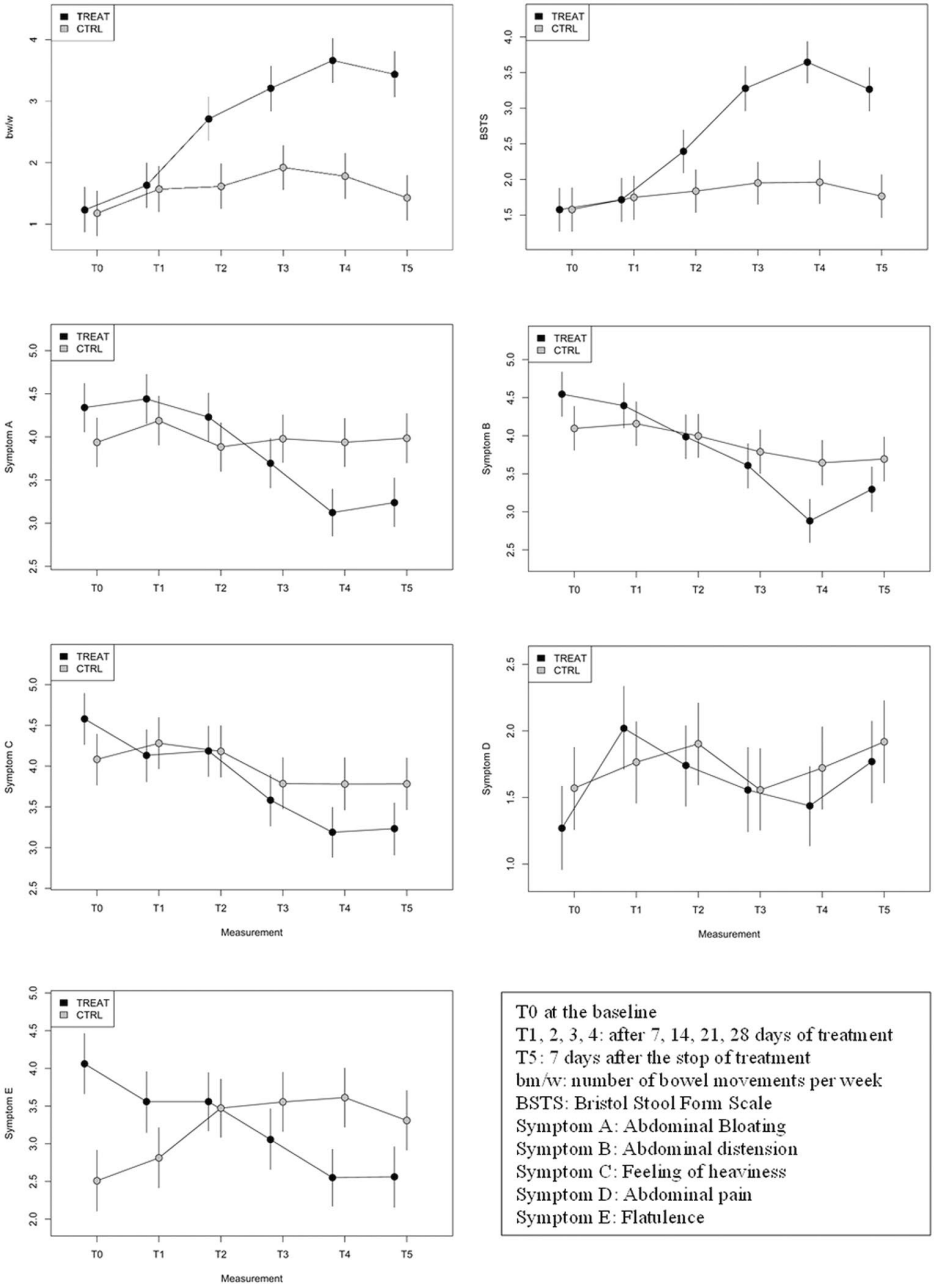
Comparison between the dietary fiber‐based formulation (TREAT) and placebo (CRT) groups in the primary and secondary outcomes measured in the clinical trial as predicted by LMM models.

The LMM for the number of bowel movements per week (Table 3) revealed a significant effect of the measurement (*p* < 0.001), treatment (*p* < 0.001), and their interaction (*p* < 0.001). This indicates that the frequency of bowel movements varied across the five measurements (T0—T5) differently in the TREAT and CTRL groups. No significant effects were observed for the participants' age or sex (Table 3). In the CTRL group (Figure 2), the weekly bowel movement frequency remained largely unchanged between consecutive measurements. However, a modest increase in the number of bowel movements was observed between T0 and T4 (*β* = 0.60 ± 0.23, t188 = 2.591, *p* = 0.010, Figure 2), which was no longer evident at the follow‐up (T0‐T5 comparison: *β* = 0.25 ± 0.23, t188 = 1.079, *p* = 0.28, Figure 2). In the TREAT group, the number of bowel movements increased progressively (Figure 2). Notably, there was a significant difference in the number of evacuations between T0 and T4 (*β* = 2.43 ± 0.23, t188 = 10.43, *p* < 0.001, Figure 2), and T0 and T5 (*β* = 2.20 ± 0.23, t188 = 9.499, *p* < 0.001, Figure 2). No significant difference was observed between groups at T0 and T1 (*β* < 0.06 ± 0.25, t188 < 0.249, *p* > 0.80, Figure 2). At T2, the number of bowel movements per week was significantly higher in the TREAT group compared to the CTRL group (*β* = 1.09 ± 0.25, t203 = 4.350, *p* < 0.001; Figure 2). This pattern continued at T3 (*β* = 1.28 ± 0.25, t203 = 5.081, *p* < 0.001; Figure 2), T4 (*β* = 1.88 ± 0.25, t203 = 7.423, *p* < 0.001; Figure 2), and T5 (*β* = 2.00 ± 0.25, t203 = 7.953, *p* < 0.001; Figure 2).

**TABLE 3:** Results of the LMMs for the comparisons between TREAT and CRTL groups for the primary (bm/w) and secondary (BSTS and Symptoms A–E) outcomes of the study.

Variable	*F*	df	*p*
bm/w			
Measurement	26.12	5188	< 0.001
Treatment	60.69	1.38	< 0.001
Sex	0.229	1213	0.63
Age	1.279	1215	0.26
Measurement × Treatment	13.43	5188	< 0.001
BSTS			
Measurement	25.70	5188	< 0.001
Treatment	62.87	1.38	< 0.001
Sex	0.014	1213	0.90
Age	2.852	1215	0.093
Measurement × Treatment	15.05	5188	< 0.001
Symptom A			
Measurement	12.41	5188	< 0.001
Treatment	1.622	1.38	0.21
Sex	0.864	1213	0.35
Age	0.031	1215	0.86
Measurement × Treatment	9.916	5188	< 0.001
Symptom B			
Measurement	22.74	5188	< 0.001
Treatment	0.872	1.38	0.36
Sex	1.273	1213	0.26
Age	0.228	1215	0.64
Measurement × Treatment	5.971	5188	< 0.001
Symptom C			
Measurement	13.76	5188	< 0.001
Treatment	2.175	1.38	0.15
Sex	0.138	1213	0.71
Age	0.004	1215	0.95
Measurement × Treatment	3.760	5188	0.0029
Symptom D			
Measurement	3.322	5188	0.0065
Treatment	1.518	1.38	0.22
Sex	1.736	1213	0.19
Age	0.946	1215	0.33
Measurement × Treatment	0.961	5188	0.44
Symptom E			
Measurement	2.434	5188	0.036
Treatment	0.005	1.38	0.93
Sex	1.411	1214	0.24
Age	1.843	1217	0.17
Measurement × Treatment	14.97	5188	< 0.001

### 3.2 Secondary Outcomes

The LMM analysis for the Bristol Stool Form Scale (Table 3) indicated significant effects for the measurement (*p* < 0.001), treatment (*p* < 0.001), and their interaction (*p* < 0.001), suggesting that the BSFS score changed differently across the five measurements (T0–T5) between the TREAT and CTRL groups. Age and sex did not have significant effects (Table 3). In the CTRL group (Figure 2), the BSFS score remained unchanged between consecutive measurements, with no significant differences between T0 and T4 (*β* = 0.38 ± 0.20, t188 = 1.956, *p* = 0.052; Figure 2) or between T0 and T5 (*β* = 0.19 ± 0.20, t188 = 0.953, *p* = 0.34; Figure 2). In the TREAT group, the BSFS increased progressively (although it slightly decreased at follow‐up measurements). There was a highly significant difference in the BSFS score between T0 and T4 (*β* = 2.07 ± 0.20, t188 = 10.46, *p* < 0.001; Figure 2) and between T0 and T5 (*β* = 1.69 ± 0.20, t188 = 8.575, *p* < 0.001; Figure 2). No significant differences were observed between groups at T0 and T1 (*β* < 0.03 ± 0.21, t213 < 0.151, *p* > 0.88; Figure 2). At T2, the BSFS score was significantly higher in the TREAT group compared to CTRL (*β* = 0.55 ± 0.21, t213 = 2.663, *p* = 0.008; Figure 2), and the same result was observed at T3 (*β* = 1.32 ± 0.20, t213 = 6.239, *p* < 0.001; Figure 2), T4 (*β* = 1.68 ± 0.20, t213 = 8.033, *p* < 0.001; Figure 2), and T5 (*β* = 1.50 ± 0.20, t213 = 7.186, *p* < 0.001; Figure 2).

Regarding symptom A (abdominal bloating), the LMM model identified significant effects only for the measurement (*p* < 0.001) and the measurement × treatment interaction (*p* < 0.001), suggesting that the intensity of abdominal bloating differed across the five measurements (T0–T5) in the TREAT and CTRL groups. Age and sex did not have significant effects (Table 3). In the CTRL group, abdominal bloating intensity remained stable across measurements. No significant differences were observed between T0 and T4 (*β* = 0.01 ± 0.17, t188 = 0.001, *p* = 0.99; Figure 2) or between T0 and T5 (*β* = 0.05 ± 0.17, t188 = 0.286, *p* = 0.77; Figure 2). In the TREAT group, abdominal bloating decreased progressively, with the intensity remaining unchanged during follow‐up. Significant differences were observed between T0 and T4 (*β* = 1.21 ± 0.17, t188 = 6.902, *p* < 0.001; Figure 2) and T0 and T5 (*β* = 1.10 ± 0.17, t188 = 6.296, *p* < 0.001; Figure 2). At T0, there was a significant difference in abdominal bloating intensity between the TREAT and CTRL groups (*β* = 0.40 ± 0.20, t188 = 2.048, *p* = 0.042, Figure 2), but no significant differences were found at T0, T1, and T2 (*β* < 0.34 ± 0.19, t188 < 1.754, *p* > 0.081, Figure 2). At T4 and T5, swelling intensity was significantly lower in the TREAT group compared to the CTRL group (T4: *β* = 0.81 ± 0.19, t189 = 4.142, *p* < 0.001; T5: *β* = 0.75 ± 0.19, t189 = 3.840, *p* < 0.001; Figure 2).

For symptom B (abdominal distension), the LMM model (Table 3) showed significant effects for the measurement (*p* < 0.001), and the measurement × treatment interaction (*p* < 0.001), indicating different patterns of symptom intensity across the five measurements (T0–T5) in the TREAT and CTRL groups. Age and sex did not show significant effects. In the CTRL group (Figure 2), abdominal distension intensity decreased significantly between T0 and T4 (*β* = 0.45 ± 0.18, t188 = 2.513, *p* = 0.013; Figure 2) and between T0 and T5 (*β* = 0.40 ± 0.18, t188 = 2.234, *p* = 0.027; Figure 2). In the TREAT group, the intensity of abdominal distension decreased progressively, with a subsequent increase during follow‐up. Significant differences were found between T0 and T4 (*β* = 1.66 ± 0.18, t188 = 9.239, *p* < 0.001; Figure 2) and between T0 and T5 (*β* = 1.25 ± 0.18, t188 = 6.982, *p* < 0.001; Figure 2). At T0, abdominal distension was significantly more intense in the TREAT group compared to the CTRL group (*β* = 0.45 ± 0.20, t186 = 2.233, *p* = 0.027, Figure 2), but there were no significant differences between treatments at T1, T2, and T3 (*β* < 0.24 ± 0.20, t186 < 1.192, *p* > 0.23; Figure 2). However, at T4 and T5, the TREAT group had significantly lower abdominal distension intensity compared to the CTRL group (T4: *β* = 0.76 ± 0.20, t186 = 3.773, *p* < 0.001; T5: *β* = 0.40 ± 0.20, t186 = 1.985, *p* = 0.048; Figure 2).

Regarding the feeling of heaviness (Symptom C), the LMM model (Table 3) revealed a significant effect for the main measurements (*p* < 0.001), and the measurement × treatment interaction (*p* = 0.0029). This indicates that the intensity of symptom C changed differently across the 5 measurement time points (T0–T5) in the TREAT and CTRL groups. In the CTRL group (Figure 2), symptom C did not show significant variation across consecutive measurements (Table 2). The intensity of symptom C remained unchanged when comparing values between T0 and T4 (*β* = −0.03 ± 0.21, t188 = 1.451; *p* = 0.15; Figure 2) and between T0 and T5 (*β* = 0.03 ± 0.21, t188 = 1.451, *p* = 0.15; Figure 2). In contrast, for subjects who took the dietary fiber‐based formulation, the intensity of symptom C progressively decreased over time and remained unchanged in the follow‐up period. There was a highly significant difference in the value of symptom C between T0 and T4 (*β* = 1.39 ± 0.21, t188 = 6.708, *p* < 0.001; Figure 2), as well as between T0 and T5 (*β* = 1.35 ± 0.21, t188 = 6.531, *p* < 0.001; Figure 2). At the T0 measurement, the intensity of symptom C was significantly higher in the TREAT group compared to the CTRL group (*β* = 0.50 ± 0.22, t213 = 2.277, *p* = 0.024; Figure 2). However, by T1, no significant differences were observed between treatments (*β* < 0.21 ± 0.22, t213 < 0.934, *p* > 0.35; Figure 2). At T4, however, the intensity of symptom C was significantly lower in the TREAT group compared to the CTRL group (*β* = 0.59 ± 0.22, t213 = 2.693, *p* = 0.0077; Figure 2), with the same result observed at T5 (*β* = 0.55 ± 0.22, t213 = 2.505, *p* = 0.013).

The LMM model for symptom D (abdominal pain; Table 3) revealed a significant effect only for the main effect of measurement (*p* = 0.0065), with no significant effect found for treatment or the measurement‐treatment interaction. This suggests that the changes in the intensity of abdominal pain were similar across the five measurement time points (T0–T5) for both the CTRL and TREAT groups. The trend in symptom D intensity fluctuated similarly across the six measurements for both the experimental groups (Figure 2). As with other symptoms, no significant effects related to the age or sex of the volunteers were observed.

The LMM model for symptom E (flatulence; Table 3) identified a significant effect for only the main effects of measurement (*p* = 0.036) and the interaction between measurement and treatment (*p* < 0.001). This indicates that the intensity of flatulence changed differently across the 5 measurement time points (T0–T5) in the TREAT and CTRL groups. In the CTRL group, the value of symptom E remained largely unchanged across consecutive measurements, with only one significant variation detected between T1 and T2 (*β* = 0.66 ± 0.26, t188 = 2.592, *p* = 0.010; Figure 2). However, the intensity of flatulence increased significantly between T0 and T4 (*β* = 1.10 ± 0.26, t188 = 4.306, *p* < 0.001; Figure 2) and between T0 and T5 (*β* = 0.80 ± 0.26, t188 = 3.132, *p* = 0.0020; Figure 2). In contrast, in subjects who took the MNS‐36, the intensity of symptom E progressively decreased with each measurement and remained stable in the follow‐up (Figure 2). As a result, a highly significant difference in the value of symptom E was observed between T0 and T4 (*β* = 1.51 ± 0.26, t188 = 5.878, *p* < 0.001; Figure 2), as well as between T0 and T5 (*β* = 1.50 ± 0.26, t188 = 5.872, *p* < 0.001; Figure 2).

As far as the treatment adherence is concerned, the total number of sachets calculated at the end of the four visits, t1 to t4, was always less than three for all recruited subjects, suggesting good compliance. Finally, none of the subjects recruited in the study took salvage treatments and no adverse events were reported to the investigator both in control and treated subjects.

## 4 Discussion

The results of this 28‐day study demonstrate that twice‐daily administration of MNS‐36, a fiber‐based formulation composed of resistant dextrin from wheat starch ( *T. aestivum* ), pectins and insoluble fibers from *Citrus* spp., and oat fibers ( *A. sativa* L.) at the daily dose of 13 g, significantly improved both the frequency and consistency of bowel movements in adults with chronic primary functional constipation. MNS‐36 is characterized by a specific fiber profile, consisting of approximately 80% soluble fiber fractions (including resistant dextrins, pectins, and β‐glucans) and approximately 20% insoluble fibers (mainly cellulose, arabinoxylans, and hemicelluloses). Although polyphenols may be naturally associated with some fiber ingredients (Xie et al. 2024), in this case their content is negligible; therefore, their contribution in this formulation is expected to be minimal and unlikely to have influenced the observed clinical effects.

Specifically, MNS‐36 increased the average number of bowel movements per week from one to more than three and normalized stool consistency from hard or lumpy to normal. This beneficial effect was statistically significant after only 2 weeks of treatment and was sustained even after a 1‐week treatment‐free period, supporting its clinical relevance. In addition, MNS‐36 was associated with a significant reduction in most constipation‐related symptoms (with the exception of abdominal pain), leading to measurable improvements in participants' quality of life.

The beneficial effects of dietary fiber supplementation on stool frequency and consistency are well‐established, particularly when integrated into a balanced dietary approach. Within the large intestine, dietary fiber exerts its laxative action through two main mechanisms. First, soluble fibers absorb water and form a gel‐like matrix that increases stool bulk and softens consistency. Second, insoluble fibers mechanically stimulate the intestinal mucosa, enhancing mucus secretion and promoting motility. Together, these mechanisms facilitate easier evacuation.

A comprehensive meta‐analysis of randomized controlled trials examining the effects of fiber supplementation on constipation in adults provided strong evidence supporting our study's findings and the efficacy of fiber in improving bowel function. This meta‐analysis included data from 473 participants, with 66% (311/473) showing a positive response to fiber supplementation (van der Schoot et al. 2022). Specifically, psyllium and pectin demonstrated notable efficacy in improving symptoms, leading to significant improvements in 41% (134/329) of participants receiving these fibers. The meta‐analysis indicated that the magnitude of improvement (in terms of stool frequency and consistency) was greatest with high doses of fiber (> 10 g/day), regardless of the treatment duration. Quantitatively, this improvement translated to a significant increase in stool frequency and stool consistency. The most substantial improvements in both frequency and consistency were observed with high doses of psyllium and pectin, particularly with treatment durations exceeding 4 weeks (van der Schoot et al. 2022). This meta‐analysis concludes that dietary fiber, particularly pectins and psyllium, exerts a significant beneficial impact on intestinal function, facilitating improved transit time and alleviating constipation symptoms in agreement with our results.

Oat fiber is well‐known for its beneficial effects on digestive health, including the alleviation of constipation (Paudel et al. 2021; Sargautiene et al. 2018; Tosh and Bordenave 2020). In a 12‐week study of 50 elderly patients with constipation, twice‐daily consumption of oat bran biscuits significantly improved bowel frequency, stool consistency, and reduced pain during defecation, without adverse effects (Valle‐Jones 1985). Another 12‐week parallel‐arm trial in frail, laxative‐dependent geriatric patients (*n* = 30; age 57–100 years) showed that oat bran (at the daily dose of 18 g, containing 8.3 g/100 g non‐digestible fermentable fiber and 9.7 g/100 g non‐digestible non‐fermentable fiber) led to laxative discontinuation in 59% (*p* < 0.001), while maintaining body weight and reducing weight loss in the control group (*p* < 0.005) (Sturtzel and Elmadfa 2008). These improvements are attributed to oat's high soluble fiber content (including β‐glucans), which increases stool consistency and promotes regular transit, its prebiotic effects fostering healthy gut microbiota, and its rich nutritional composition (essential amino acids, protein, fat, avenanthramides) and potential anti‐inflammatory actions, all contributing to improved bowel function (Kumar et al. 2021; Paudel et al. 2021; Peterson 2001; Sadiq Butt et al. 2008; Sargautiene et al. 2018; Tosh and Bordenave 2020).

Pectins, like other dietary fibers, exhibit numerous beneficial physiological effects, including slowing gastric emptying, improving bowel function, reducing glucose and cholesterol absorption, and increasing fecal mass. These properties make pectins a promising component in both food science and medicine (Zaitseva et al. 2020). Structurally, pectin polysaccharides (PPS) are complex and variable, characterized by an irregular arrangement of constituent carbohydrates. PPS forms part of the cell wall in nearly all higher plants, with galacturonic acid residues present as either methyl esters or salts. In the primary cell walls of dicotyledonous and monocotyledonous plants, pectins account for about 35% of the total content, playing a critical role in plant structure and water retention (Zaitseva et al. 2020).

The efficacy of pectins in managing intestinal transit and constipation was demonstrated in a randomized, controlled trial involving 80 adults with slow colonic transit constipation. Participants received either 24 g/day of pectin (fiber group) or maltodextrin (placebo group) for 4 weeks. Measurements of colonic transit time (CTT), constipation symptoms, and fecal bacterial populations before and after treatment revealed significant improvements in the fiber group. Post‐treatment CTT was significantly shorter in the fiber group compared to both its pre‐treatment levels (60.2 ± 11.2 h vs. 80.3 ± 9.5 h, *p* < 0.01) and the placebo group (79.4 ± 11.7 h, *p* < 0.01). The constipation score in the fiber group also decreased significantly compared to its pre‐treatment levels and the placebo group (*p* < 0.05). Additionally, beneficial gut bacteria, such as Bifidobacterium sp. and Lactobacillus sp., increased markedly (*p* < 0.05), while Clostridium sp. decreased significantly (*p* < 0.05). No significant adverse effects were reported. These findings indicate that a 4‐week pectin regimen can accelerate colonic transit time, alleviate constipation symptoms, and promote healthier gut microbiota (Zaitseva et al. 2020).

The benefits of dietary fiber, especially pectins, for improving bowel transit time and intestinal activity are well established (van der Schoot et al. 2022). A recent double‐blind, randomized, placebo‐controlled study investigated the effects of prune juice, which contains pectins and polyphenols, on Japanese individuals with functional constipation. Consuming 54 g/day of prune juice for 8 weeks significantly decreased the incidence of hard, lumpy stools, increased normal stool frequency, and did not lead to diarrhea or loose stools. Furthermore, prune intake improved subjective complaints of constipation without causing flatulence, urgency, or adverse effects on liver or kidney function (Koyama et al. 2022).

Regarding the mechanism of action through which dietary fiber exerts its beneficial effects on constipation, preclinical studies have explored the potential of resistant dextrins as prebiotic agents, particularly their effects on intestinal microbiota and fecal enzyme activity. One study investigated resistant dextrin derived from potato starch, focusing on its influence on the growth of specific bacterial cultures (*Lactobacillus, Bifidobacterium, Escherichia coli, Enterococcus, Clostridium*, and *Bacteroides* spp.) in media enriched with resistant dextrin. The findings demonstrated that resistant dextrin serves as an effective carbon source for gut bacteria, promoting the growth of beneficial strains such as *Lactobacillus* and *Bifidobacterium* spp. while inhibiting the proliferation of other intestinal strains (*Clostridium*, *E. coli*, *Enterococcus*, and *Bacteroides* spp.). After 168 h of culture, probiotic strains exhibited increased viability, and a high prebiotic index was observed, with statistical significance (*p* < 0.05). Interestingly, the presence of resistant dextrin resulted in no significant change in overall culture pH; however, the rate of pH decrease was slower compared to control conditions. Resistant dextrin also influences fecal enzyme activities. It significantly reduced the activity of β‐glucosidase and β‐glucuronidase compared to controls (*p* < 0.05), while the impact on other enzymes was less pronounced. These findings highlight the potential of resistant dextrin as a prebiotic agent capable of enhancing the growth of beneficial gut bacteria, modulating enzyme activities, and maintaining a stable gut environment (Wlodarczyk et al. 2022).

The production of resistant dextrin from partially hydrolyzed wheat starch involves a controlled hydrolysis process, where starch chains are broken down into smaller molecules, such as dextrins, using enzymatic or chemical treatments. These dextrins are then subjected to heat treatment to render them resistant to digestion in the small intestine. This resistant dextrin can be incorporated into food products as a fiber‐enriching ingredient or can be used as food supplement ingredient. Resistant dextrins are particularly promising as prebiotic agents because they encourage the growth of beneficial gut bacteria and positively influence health indicators, such as fecal enzyme activity (Wlodarczyk et al. 2022).

A clinical study examined the effects of continuous resistant dextrin administration on intestinal gas production, digestive activity, metabolism, and gut microbiota composition. Twenty healthy participants received 14 g/day of resistant dextrin for 4 weeks. Initially, resistant dextrin consumption led to an increase in intestinal gas production and associated discomfort, which subsided over time. Following withdrawal, gas production decreased further. Resistant dextrin expanded colon biomass volume, induced changes in metabolism, and altered microbial composition, notably increasing short‐chain fatty acid‐producing species and modulating bile acids. These findings suggest that soluble fiber consumption induces adaptive changes in gut microbiota, steering fermentation pathways towards reduced gas production (Barber et al. 2022).

The present trial adds to this body of evidence by showing that the combination of resistant dextrin, pectins and insoluble fibers from citrus, and oat fibers can provide rapid and clinically meaningful relief from constipation and its symptoms in a population with chronic functional constipation at a daily dose of 13 g, lower than that of each type of dietary fiber able to produce the same effects and without the initial discomfort induced by quite the same daily dose (14 g) of resistant dextrin. Future studies should explore whether MNS‐36 exerts sustained effects beyond the treatment period and whether improvements in bowel function are linked to measurable changes in gut microbial composition. Although the beneficial effects were clearly observed during the supplementation period, their partial reduction after treatment discontinuation suggests that continued intake may be necessary to maintain the observed improvements over time.

Despite these promising findings, several limitations should be acknowledged. The relatively small sample size, which may reduce the statistical power for some secondary analyses and. In addition, The single‐center design of this study limits the generalizability of the findings and may not fully capture the variability of the broader population, thus requiring confirmation in multicenter settings. Another important limitation is the relatively short duration of the intervention (28 days), which does not allow the assessment of long‐term effects or the persistence of the observed benefits over time. Moreover, questionnaires, particularly patient‐reported outcomes (PROs), are valuable tools for assessing symptom impact and severity. When administered during follow‐up visits, they can help determine treatment responses. The EFSA guidelines recognize their usefulness (EFSA Panel on Dietetic Products and Allergies 2016); however, it is important to remember that these questionnaires reflect subjective opinions and may be influenced by bias. Finally, although participants were instructed to maintain their usual dietary habits, the potential influence of uncontrolled dietary or behavioral factors cannot be entirely excluded. Therefore, further studies with multicenter designs, and longer follow‐up periods are warranted to confirm and extend these findings.

In conclusion, MNS‐36, when administered twice daily for 28 days at daily dose of 13 g, effectively normalized bowel movement frequency and stool consistency and reduced several constipation‐associated symptoms in individuals with chronic functional constipation. These results highlight the potential of MNS‐36 as a well‐tolerated, non‐pharmacological approach for managing constipation, particularly in patients seeking alternatives or adjuncts to conventional laxatives. Further multicenter trials with longer follow‐up and broader populations are warranted to confirm these findings and evaluate their generalizability.

## Author Contributions

**Roberto Piccinocchi:** software, validation, resources. **Hesham R. El‐Seedi:** writing – review and editing. **Alessandro Di Minno:** conceptualization, methodology, investigation, writing – original draft, writing – review and editing, visualization. **Costanza Riccioni:** formal analysis. **Danaè Larsen:** methodology, formal analysis. **Alessandra Baldi:** investigation, data curation. **Angela Cerqua:** software. **Lorenza Francesca De Lellis:** methodology, software, validation, formal analysis, data curation, writing – original draft. **Maria Vittoria Morone:** methodology, validation, data curation. **Daniele Giuseppe Buccato:** methodology, formal analysis, investigation, resources, data curation, writing – original draft. **Antonietta di Guglielmo:** resources. **Gaetano Piccinocchi:** resources, writing – review and editing. **Maria Daglia:** conceptualization, writing – original draft, writing – review and editing, supervision, project administration. **Hammad Ullah:** methodology, writing – review and editing. **Roberto Sacchi:** software, writing – original draft.

## Funding

This research received no external funding.

## Ethics Statement

This study was carried out in accordance with the current Declaration of Helsinki, concerning medical research on humans (Helsinki 1964, amended by: Tokyo 1975, Venice 1983, Hong Kong 1989, Somerset West 1996 and Edinburgh), and the Guidelines for Good Clinical Practice (CPMP/ICH/135/95), and approved by the Campania 1 Ethics Committee (protocol no. 134, 13 May 2024—N. Reg. 1–2024).

## Consent

Informed consent was obtained from all subjects involved in this study.

## Conflicts of Interest

A.D.M. was employed by CEINGE‐Biotecnologie Avanzate. C.R. is an employee of ESSERRE srl. None of the academic researchers listed as co‐authors served as consultants for ESSERRE srl or received any personal compensation from ESSERRE srl.

## Data Availability

The data that support the findings of this study are available from the corresponding author upon reasonable request.
